# Multifaceted research impacts of a large community engaged consortia: Results and methods from the RADx-UP initiative

**DOI:** 10.1017/cts.2026.10805

**Published:** 2026-07-27

**Authors:** Amelia R. DeFosset, Tara Carr, Bernard A. Coles, Marlena L. Kuhn, Kristen D. Witkemper, Barrie E. Hayes, Caroline Taheri, Abisola Osinuga Snipes, Nisha Baral, Valerie Lucas, Gaurav Dave, Leah Frerichs

**Affiliations:** 1 Abacus Evaluation, Center for Thriving Communities, Department of Social Medicine, School of Medicine, The University of North Carolina at Chapel Hill, Chapel Hill, NC, USA; 2 Health Sciences Library, The University of North Carolina at Chapel Hill, Chapel Hill, NC, USA; 3 Division of Internal Medicine, Department of Medicine, School of Medicine, University of North Carolina at Chapel Hill, Chapel Hill, NC, USA; 4 Department of Health Policy and Management, Gillings School of Global Public Health, The University of North Carolina at Chapel Hill, Chapel Hill, NC, USA

**Keywords:** COVID-19 testing, research impact evaluation, bibliometric analysis, content analysis, social network analysis

## Abstract

**Introduction:**

The Rapid Acceleration of Diagnostics-Underserved Populations (RADx-UP) initiative sought to catalyze timely scientific understanding of approaches to address COVID-19 disparities through community engaged research. It also sought to foster sustainable community-academic partnerships as key infrastructure for responding to emerging public health crises.

**Methods:**

We integrated bibliometric, Altmetric, and network analyses to examine the volume and scope of research produced by the RADx-UP initiative, its translational impacts, and how community partners were integrated into research networks. We identified Scopus- and PubMed-indexed scientific manuscripts supported by RADx-UP funding through June 2024. We extracted metrics from Scopus, PlumX, and iCite, and via structured coding. Network analysis explored community partners’ positioning in co-author networks across three time points.

**Results:**

The initiative produced 334 publications during the study period. Studies most frequently focused on Hispanic/Latino (25.1%), children and adolescents (19.2%), and Black/African American (18.9%) populations. The average relative citation ratio (RCR) was 2.07. Altmetrics indicated public engagement through mentions in earned media (761), social media (1,971), and citations in policy documents (90). Community partners were represented in 18% of network components in 2022 and 41% by 2024. While community partners’ total direct connections to other investigators did not consistently increase, their connections to influential investigators, as measured by power centrality, did (−0.96, −0.75, −0.37 in 2022, 2023, and 2024, respectively).

**Conclusions:**

These findings point to the greater-than-average impact of RADx-UP publications and initial community translation. They also highlight contributions to the research ecosystem that could scaffold long-term research quality and productivity, and broader impact.

## Introduction

The COVID-19 pandemic reflected and exacerbated existing health inequities in the United States, with members of underserved and vulnerable communities bearing a disproportionate burden of morbidity and mortality [1–6]. Driving these inequities were structural and systemic factors, such as inadequate access to diagnostics and justified mistrust in the medical establishment, resulting in lower detection and control of COVID-19 [7,8,9,10]. To mitigate harm to these communities, the National Institutes of Health (NIH) made an unprecedented investment over five years to fund the Rapid Acceleration of Diagnostics-Underserved Populations (RADx-UP) Program, a consortium of over 160 community engaged research projects across the United States (US), US territories, and Tribal Nations [5,6]. The goals of the RADx-UP program were two-fold; first to increase testing in communities with a focus on those experiencing COVID-19 disparities; second to catalyze scientific understanding of what works to prevent and address diagnostic disparities during public health crises. To achieve these aims, the program sought to foster community-academic partnerships and support them to inform the scientific direction and implementation of their individual research projects. Beyond increasing scientific output, the program sought to reshape the research ecosystem to better support community engaged work as key infrastructure for preventing and responding to emerging public health crises in the future [11,12].

Multi-faceted measures are needed to evaluate how the RADx-UP program met these multi-level goals around scientific impact. In recent years, measures and methods for assessing research impacts have proliferated. A common approach, including among large consortia research programs, is the use of bibliometric indicators, such as publication and citation counts, to measure research productivity and the influence of that research on the wider evidence base [13,14,15]. However, bibliometric analyses are limited in that they do not speak to how new knowledge diffuses to and impacts communities through on-the-ground changes to practices, policies, and conditions. Altmetrics, a relatively new class of research impact measures, seeks to address this gap by assessing initial indicators of translation, including mentions of research in social and earned media, and policy and clinical practice documents, among other areas of the gray literature [16,17,18]. This method is especially well-suited for evaluating community engaged research, as an explicit goal of this paradigm often is to accelerate dissemination beyond scholarly channels, requiring nuanced methods to accurately capture such products [19]. Additionally, social network analysis, which examines the nature of networked connections [20], has a role in examining the extent to which the RADx-UP initiative meaningfully or sustainably integrated community partners in research networks. The layering of bibliometrics and network analytic methods has previously been applied to explore the relationship between scholarly fields [19] and evaluate the online dissemination of health information [21,22,23]. Although used together in other applications, to our knowledge, this suite of methods has not been previously combined to evaluate the success of community engaged research programs.

This article addresses that gap by presenting results of a multi-faceted research impact evaluation of the RADx-UP initiative. Specifically, it sought to understand the volume and scope of research produced by the RADx-UP initiative, its initial translational impacts, and how community partners were integrated in investigative networks. By layering complementary methods that make use of publicly accessible data, this method provides an efficient and replicable model to examine the progress and effectiveness of large-scale community engaged research programs, an important and emerging research paradigm.

## Materials and methods

### Background

Abacus Evaluation, an evaluation consultancy within the University of North Carolina at Chapel Hill, conducted a comprehensive mixed methods evaluation of the RADx-UP Program, including its research impacts. The research impact evaluation occurred between 2020 and 2024, with evaluation goals and activities defined collaboratively with the RADx-UP Coordinating Center and program leadership.

### Sample

This study analyzed scientific manuscripts supported by RADx-UP funding from September 26, 2020 (start of the RADx-UP award) through June 30, 2024 (evaluation data cut off). Publications were identified in two ways. First, we conducted automated searches using variously formatted versions of the project grant number in Scopus and PubMed. Scopus is a database of peer-reviewed literature that tracks citations, maintained by Elsevier [24]. PubMed is a database of peer-reviewed literature focusing on biomedical and life sciences research, maintained by the U.S. National Library of Medicine [25]. Second, RADx-UP project staff completed quarterly evaluation surveys where they were asked to list publications since the last data collection cycle. We used this information to confirm and augment database searches. Specifically, if a publication was not returned by the automatic query, then we performed a direct search in each database with the Digital Object Identifiers. If located, the publication was added to the sample.

### Metrics and data extraction process

Once we identified our analytical sample, we used a multi-step process to collate research productivity and impact metrics across three databases: Scopus, iCite, and PlumX. Table 1. describes these metrics, their definitions, and sources. In addition to its indexing function, the Scopus database provides total citation counts, a study metrics which we directly exported for each article in our sample. The iCite database, NIH Office of Portfolio Analysis’s web-based tool, generates other metrics specifically for PubMed-indexed journal publications. iCite’s index covers work published 1980 to present [26]. For the sample publications identified in PubMed, we imported PubMed Unique Identifiers into iCite and exported relevant metrics. Scopus also provides access to PlumX metrics, which measure how the public has been interacting with scholarly articles online, from the number of times an article was downloaded to the number of times an article was shared on social media platforms. Scopus organizes these metrics into the following categories: captures, non-scholarly citations, earned media mentions, social media mentions, and usage (Table 1). We used the DOIs of publications identified using Scopus and PubMed to retrieve PlumX metrics for these publications using the Scopus PlumX Metrics Application Programming Interface.

**Table 1.** Research productivity, scope, and translation impact metrics of interest Table 1 long description.

Metric	Definition	Data source
**Research productivity and scope**		
Publications by year	The cumulative number of publications produced by year, with year defined as the calendar year when the manuscript was first made publicly available, electronically or in print.	Scopus, PubMed
Publications by study setting	The cumulative number of publications by study setting, with setting categories constructed according to RADx-UP internal operational definitions. Not all publications had a designated setting. For instance, systematic reviews and letters to the editor were not assigned a setting.	Structured coding
Publications by focal population	The cumulative number of publications reporting results of studies focused on that population, categorized based on NIH definitions of underserved and vulnerable populations [27]. Publications could have more than one focal population or no focal population.	Structured coding
Count of publications by study type	The cumulative number of publications categorized by study type, with definitions of study types developed through reference to established definitions for qualitative and quantitative research methods [28–30].	Structured coding
Co-author affiliations	The listed affiliation for publication co-authors, categorized according to an internally developed taxonomy that was vetted with the RADx-UP program team. Categories were: **Community**: Organization with direct relationships with the target population, serves target population at the community level; **School**: Formal school settings for children from pre-primary to secondary school, includes daycare, pre-school, and K-12 school; **Government**: Government organization at the local, state or federal level, including health department or other governmental organization; **Professional**: Professional groups which include professional associations, consulting groups, contract research organizations, for-profit businesses, insurance companies; **Healthcare**: Organization with a primary scope of work that includes providing health services to individuals, includes non-research hospital units and clinics; **Academic**: Conducts research within a university or hospital. When needed, a hierarchy was applied, with the highest-ranking category being selected when more than one affiliation was listed: 1) community, 2) school, 3) government, 4) professional, 5) healthcare, 6) academic.	Structured coding
**Initial translational impact**		
Scholarly citations	The number of times a publication has been cited in other scholarly publications indexed in the Scopus database.	Scopus
Relative citation ratio (RCR)	A publication′s average number of citations per year is divided by the citation rate of NIH-funded publications in the same field and year. The co-citation network, which includes all the publications cited alongside the scholarly publications, defines the field for each scholarly publication. A publication with an RCR of 1.0 has received the same number of citations per year as the typical (median) NIH-funded publication in its field. A publication must be published for at least a year or have accrued at least five citations to calculate an RCR [26].	iCite
Captures	Count of saves, archives, or other actions allowing someone to come back to a publication later. Examples include bookmarks, code forks, favorites, followers, readers, watchers, exports/saves and subscribers. Captures can be a leading indicator of future citations [31].	PlumX
Non-scholarly citations	The number of times a publication has been cited in non-scholarly documents such as patent applications, clinical practice guidelines, or legislative filings or position papers. Complementing the number of scholarly citations alone, this metric gives a sense for societal impact [31].	PlumX
Earned media mentions	Number of times a publication is cited in news or non-scholarly communication channels. Examples include blog mentions, news media mentions, reviews, and Wikipedia references. Mentions are an indicator that people are engaging with the research [31].	PlumX
Social media mentions	The number of times a publication is referenced on Facebook. Examples include comments, shares, likes, ratings, and recommendations that reference the research. This metric is an indicator of the grass-roots attention the research is receiving [31].	PlumX
Usage	The number of interactions with a publication indicating real-time use, including abstract views, clicks, downloads, full text views, library holdings, and video plays [31].	PlumX

We exported all data into Excel for further cleaning and analysis, applyingthe same cut-off date of June 30^th^, 2024. We also manually extracted and coded data elements on study and co-author characteristics captured in the publications (See Table 1). These data elements were extracted through review of the publication texts via a structured coding process completed by trained reviewers. We used consensus coding with regular quality checks during the first round of data extraction to calibrate and finalize the coding process, which we then applied on the full dataset. Table 2. describes the social network measures used for this study, their definitions, and how they were calculated [20]. We selected a set of parsimonious network measures most suited to answer our research questions. Specifically, we were interested in overall connections (node degree, here representing collaborations as co-authors) between individuals, expecting they would grow over time as productivity and cross-pollination increased across the RADx-UP program. To further operationalize community involvement, we also examined two measures of how community partners were positioned in the network, including representation across groups of collaborators (clusters) and power centrality which quantifies connections to other well-connected members in the network who might function as brokers of information or resources. In a successful community-engaged research program, we would also expect to see these metrics increase over time as community partners became meaningfully integrated in research activities.

**Table 2.** Social network measures of interest Table 2 long description.

Measure	Definition	Level
Node degree	The number of direct connections a node (i.e., member) has. In this context it is the number of co-authors an author has across their publications.	Individual
Components	A cluster of nodes with connections to each other and no connections to nodes outside their cluster.	Network
Community representation	Percent of components containing co-authors who either represent community organizations or have strong affiliations with one.	Network
Power centrality	A common measure of influence in social network analysis where the centrality of individuals changes based on their connection to other nodes and their respective influence in the network. Positive values of power centrality imply that members of the network are connected to relatively central members occupying a position important to the flow of information. Conversely, negative values reflect connection to members who are relatively less central.	Individual

### Analysis

We analyzed publication data descriptively to explore research productivity and scope across RADx-UP funded projects. Analysis occurred in Microsoft Excel and R version 4.4.2 [32,33]. Social Network Analysis descriptively explored the metrics of interest, using extracted and coded co-author data to derive both measures of individual connectivity and position (individual-level measures) and the characteristics of the network as a whole (network-level measures) (Table 2). We created network maps to visualize collaboration across the RADx-UP investigator community overall, and the representation and positionality of community co-authors over time. To produce the maps, we created force-directed layouts in R that place strongly connected nodes closer together and weakly connected or disconnected nodes further apart. We calculated metrics and visualized the network at three time periods using standard annual data cut-offs (March 2022, 2023, and 2024). All analyses, including visualizations, were cumulative, accounting for all publications available at the time of the data cut-off. We selected 2022 as the first analysis year to allow time for RADx-UP programming to influence collaborations, as collaborations observed in the first grant year (2020–2021) were more likely to be resultant from prior relationships.

## Results

### Research productivity and scope

The RADx-UP initiative produced a total of 334 publications during the analysis period, which began in late 2020. The annual rate of publications increased over time, peaking in 2022 and then dropping slightly the following year. Although publications were only tracked through June 2024, available data suggest the rate of publications in that year was similar to 2022 and 2023. Table 3 further characterizes the scope of this body of literature. Among publications reporting study results with a discrete setting (136 in total, 41% of publications), the most reported setting was schools (18.6%). Approximately a quarter of publications reported results of studies focused on Hispanic/Latino populations. Children and adolescents (19.2%), Black or African Americans (18.9%), individuals with low socio-economic status (13.2%) and rural communities (10.8%) were the next most reported focal populations. A strong majority of publications (86.9%) were research articles. Of these, 67.2% reported results of quantitative, 17.9% qualitative, and 14.8% mixed methods analyses.

**Table 3.** Scope of publications produced by the RADx-UP initiative through June 2024 (*n* = 334) Table 3 long description.

Publication characteristics	Count (%) of publications
Publication year	
2020	1 (0.3)
2021	42 (12.6)
2022	125 (37.4)
2023	109 (32.6)
2024	57 (17.1)
Setting^1^	
School	62 (18.6)
Residential care facility	22 (6.6)
Community health center	22 (6.6)
In-home	12 (3.6)
Hospital	11 (3.3)
Prison or jail	7 (2.1)
Focal population^2^	
Hispanic or Latino	84 (25.1)
Children or adolescents	64 (19.2)
Black or African American	63 (18.9)
Low socioeconomic status	44 (13.2)
Rural	36 (10.8)
People who inject drugs	26 (7.8)
American Indian	23 (6.9)
Older adults	22 (6.6)
Asian Americans	17 (5.1)
Pacific Islanders	12 (3.6)
People who are incarcerated	11 (3.3)
Immigrants or refugees	11 (3.3)
Alaska Natives	9 (2.7)
People experiencing homelessness	8 (2.4)
Native Hawaiian	8 (2.4)
Pregnant people	6 (1.8)
Sexual and gender minorities	5 (1.5)
Study type	
Original research	290 (86.9)
*Quantitative*	*195 (67.2)* ^3^
*Qualitative*	*52 (17.9)* ^3^
*Mixed methods*	*43 (14.8)* ^3^
Editorial	18 (5.4)
Literature review	13 (3.9)
Clinical case study	4 (1.2)

1 Only includes publications with a clearly designated setting. Percentages do not add to 100.

2 Publications could be coded as having more than one focal population. Percentages do not add to 100.

3 Denominator is number of original research articles, *n* = 290.

### Initial translational impact

At the time of the study, RADx-UP publications had amassed 2,641 total citations, or about eight per publication (Table 4). However, there was significant variation across studies and citation counts are time-varying (i.e., a publication from 2024 would not be expected to have as many citations as one from 2022). Eight RADx-UP publications received 60 or more citations during the study period. The average RCR was 2.07, with 1.0 being the median citation rate for NIH-funded papers in the corresponding publication year and field. While there were over 8,000 captures, a metric indicating potential future citation or use, indicators of real-time usage were considerably lower (122 downloads, 115 abstract views). In addition to citations in the scholarly literature, there were 90 non-scholarly citations, all of which were in policy documents (e.g., legislative filings or position papers). There were just over 750 earned media mentions, including references in print and broadcast news and blogs, and close to 2,000 mentions of publications on Facebook.

**Table 4.** Translational impact metrics for publications produced by the RADx-UP initiative through June 2024 (*n* = 334) Table 4 long description.

Metric	Mean (range)
**Bibliometrics**	
Total citation count (*n* = 328)^1^	2,641 (0–91)
Relative citation ratio (*n* = 300)	2.07 (.00–12.94)
**Altmetrics (plumX)**	
Captures	8,208
Usage	
Downloads	122
Abstract views	115
Non-scholarly citations	
Policy citations	90
Earned media mentions	
News	736
Blogs	25
Social media mentions	
Facebook	1,971

1 Includes self-citations. Total without self-citations is 2,084.

### Integration of community partners

**Figure 1.**
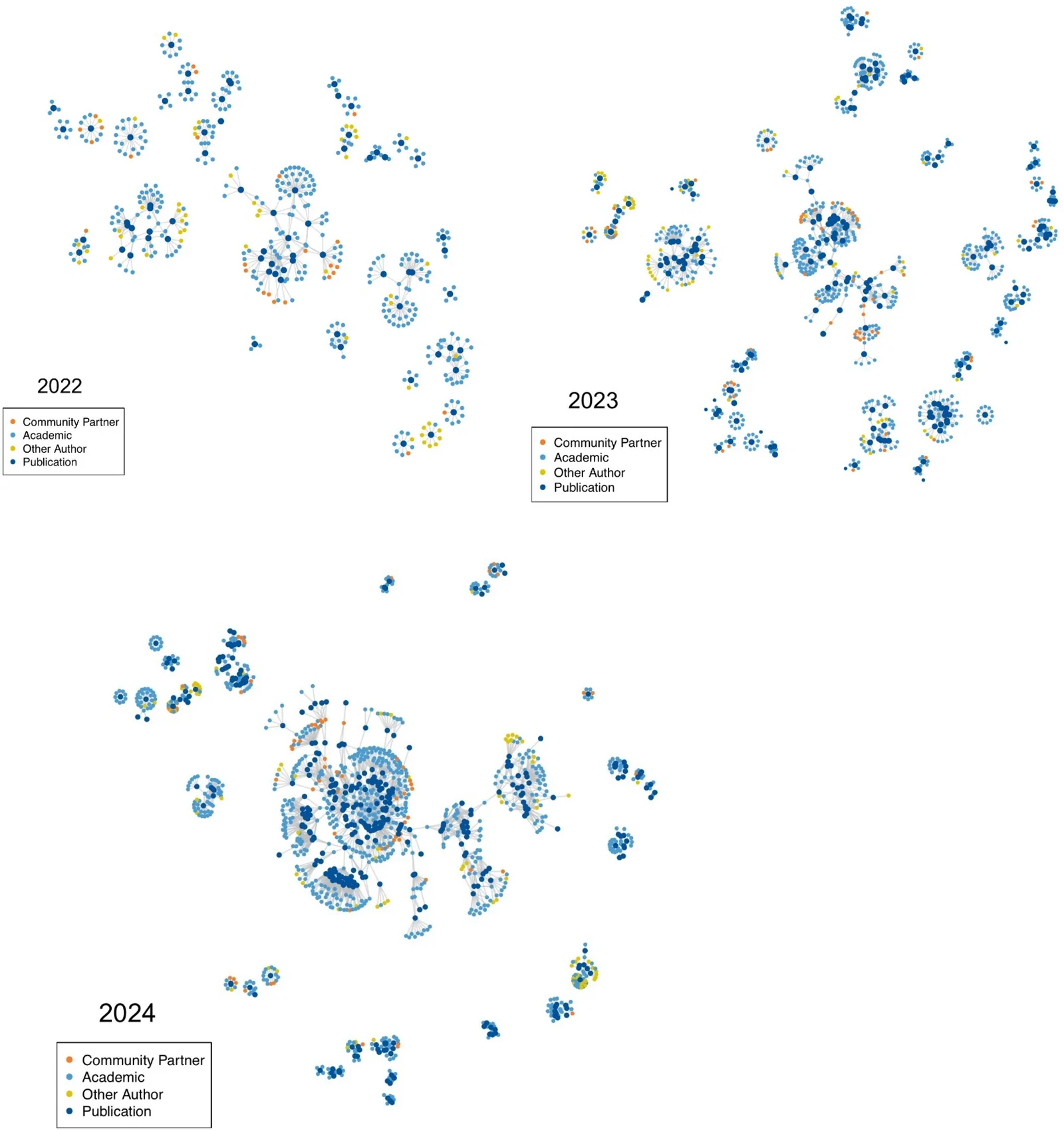
Community partner integration into the RADx-UP publications co-author network, 2022–2024.

Table 5 reports descriptive statistics for the authorship network across RADx-UP funded publications during the study period, Figure 1 visualizes the network in 2022, 2023, and 2024. Across all author types, the average node degree, or the number of direct connections (represented by lines) an author (represented with dots) had with other authors in the network, increased by 3.67 between 2022 and 2023, and 7.98 between 2023 and 2024. Across years, we see the number of authors in the network increase, alongside the connections between them (Figure 1). Median Degree similarly increased year-over-year, with 50% of authors collaborating with more than 15 other authors in 2024, up from the median of 11 authors in 2022. There were larger increases in author collaboration between 2023 and 2024 than between 2022 and 2023.

**Table 5.** Network analysis metrics for the RADx-UP publications co-author network, overall and for community partners Table 5 long description.

Metric	2022	2023	2024
Network overall			
Mean node degree	14.37	18.04	26.02
# of components	28	34	29
Community partners			
Representation in components	.18	.37	.41
Mean node degree	13.83	25.17	19.69
Power centrality	−0.96	−0.75	−0.37

Network components are groups of nodes that share direct connections to each other but are otherwise disconnected from the rest of the network. In the context of this study, they illustrate distinct research communities where scholars often collaborate. Across all years, these groups ranged greatly in size with the smallest components containing only a single publication and its associated authors while the largest component housed 166 publications and 425 authors across 47 RADx-UP funded publications. In 2022 there were 28 network components, which increased to 34 in 2023, before dropping to 29 in 2024. As evidenced by Figure 1, this drop was a result of new connections forming between formerly disconnected components as connectivity increased overall across the network and a single large author community formed by 2024.

Representation of community partners in the network (shown with orange dots, Figure 1) increased steadily over the study period. In 2022, 17.86% of clusters contained community partners, which increased to 36.84% and 41.38% in 2023 and 2024 respectively. The mean node degree for community partners, i.e. the average number of co-authors community partners collaborated with, increased initially from 13.83 (lower than average for all co-authors) to 25.17 (higher than full-group average), before dropping in 2024 to 19.69. Over all years, community partners had negative power centrality values, reflecting that relative to others in the network they were connected to less central members. However, the average power centrality of community partners increased across all three years, reflecting a steady positive trend in connection to central members.

## Discussion

The COVID-19 crisis was unprecedented and highly dynamic, with existing literature holding few parallels to drive strategy development, especially as emerging disparities required community-specific solutions. Rapid dissemination of a wealth of research findings, then, was critical. Our findings suggest RADx-UP largely delivered on this goal by producing a large, diverse, and timely literature base. The annual rate of publications peaked in 2022, with over half of all publications released while the national public emergency declaration was still in effect [34]. Targeting research towards communities disproportionately impacted by COVID-19 was also a foundational goal. This study highlights how the RADx-UP initiative produced research focused on addressing disparities in the Hispanic/Latino and Black/African American communities, two populations with disproportionate infection rates and/or severity [35,36], while highlighting potential missed opportunities. Specifically, relatively fewer publications explicitly focused on low socioeconomic status or rurality (which is strongly associated with healthcare access) [37,38], although evidence points to distinct interactions between these factors, race/ethnicity, and COVID-19 outcomes [39].

Bibliometric results provide evidence of initial translational impact of the RADx-UP body of literature. Time- and field- normalized RCR scores indicate the RADx-UP publications have a greater-than-typical citation rate compared to NIH-funded papers in the same field and year, ergo a greater-than-typical scientific influence compared to peer publications. Public engagement with RADx-UP publications, as indicated by Altmetric data was notable, especially in the form of captures, mentions in earned and social media, and citations in policy documents. As a counterpoint, usage (an indicator of deeper engagement) was relatively low. Citations in policy documents, in particular, speak to translation, as they demonstrate not just momentum, but actual influence within health legislation. One interpretation of these finding is that the RADx-UP initiative was speaking to a critical evidence gap, as intended and noted above. It’s also worth investigating further the role community engagement played in increasing the credibility of, and interest in, this literature base. Proponents of community engaged research theorize that this paradigm both generates higher quality science and makes it more trustworthy, relevant, and ultimately impactful [40,41]. In applying a community engaged lens, the RADx-UP initiative had an intentional strategy to share findings with the public through multiple channels, including social media. Our findings help to monitor the success of these efforts while lending some credence to the notion that community engaged research can have heightened impact, although further research is needed.

Network analysis findings support that community partners became more integral within RADx-UP scientific community as time went on. As the investigator network overall grew and became more connected, representation of community partners also grew, with close to half of all components including community partners by 2024. Interestingly, while the individual connections of community partners did not consistently increase, their connections to influential investigators did. It’s possible that this proximity is indicative of their perspectives and priorities having meaningful influence on the body of literature. There could also be implications for the sustainability of these partnerships and their longer-term impacts. It’s reasonable to assume community partners may have less dedicated time to engage in research, or comparable career-related motivations, relative to academic partners. Being able to see the concrete outcomes of their participation, including influence over research output, future research directions or translational impact, may be critical to motivate continued involvement [42]. Additionally, influential actors in the network are more likely to be brokers of resources, like future grant funding [20], and direct connections to these investigators could facilitate continued partnerships, building on and extending from the RADx-UP initiative. These findings add nuance to our understanding of the research impact of the RADx-UP initiative, highlighting changes to the research ecosystem that could scaffold long-term research quality and productivity, and broader impact.

## Limitations and future directions

This analysis has several limitations that should be considered when interpreting results. First, although we used a multi-step process to identify and extract data for eligible publications, it’s likely data are incomplete as not every publication was indexed in relevant databases. We were also limited to the metrics indexed in our source databases or otherwise publicly available. In some cases, this information offered incomplete coverage for the constructs of interest. For instance, PlumX only tracks mentions in Facebook as an indicator of social media mentions which may not cover the breadth of engagement with health topics online or may skew towards certain demographics. Information about co-author contributions (e.g., Principal Investigator status, roles in manuscript development) could have added to the analysis of co-author networks and community integration but were not reliably available across publications. Relatedly, data are point-in-time, and some metrics (i.e., citation counts) are sensitive to temporal factors. Reporting multiple metrics, especially time-normalized RCR, helps to address this limitation. Nevertheless, using a longer time horizon would have given a fuller sense of research impact. Indicators used in this study reflect the earliest possible signals of potential real-world translation, which is directly measured through changes in clinical practice, policy landscapes, and community, and economic conditions [43]. These changes take time to accumulate and are complex to measure. As a first step, an exploration of the ways in which RADx-UP publications were cited in policy documents, which was out of scope for the present analysis, could further characterize translation.

As the field of bibliometrics analysis, and use of Altmetrics, is emerging, data that could be used to benchmark study results relative to comparable research initiatives were not available. The network analysis shares a similar limitation, as there are no empirically established standards around the level or nature of community partner involvement linked to meaningful or sustainable research partnerships. Developing these benchmarks and integrating them into future bibliometric or network analytic studies could optimize the power and utility of these methods to drive decision making. Additionally, examining causal relationships between characteristics of individual studies (e.g., level of community engagement, study type) and translational impact was beyond the scope of this study; any reflections regarding these relationships are purely speculative and highlight the need for additional research in this area. Finally, this study leveraged publicly accessible secondary data (available with and without subscription), an efficient approach to assess multiple levels of research output and impact. That said, collecting primary data would have allowed deeper and more direct examination of some of the issues of interest in this study, including the nature of relationships between co-authors and the influence of relationships predating the RADx-UP initiative. Future studies that engage community-academic co-authors to characterize their relationships and assess the long-term trajectory and outcomes of these relationships would enrich the literature base around community engaged research.

## Table 1. Long description

A table with four columns and twelve rows. The columns are labeled Metric, Definition, and Data source. The rows are labeled Research productivity and scope, Publications by year, Publications by study setting, Publications by focal population, Count of publications by study type, Co-author affiliations, Initial translational impact, Scholarly citations, RCR, Captures, Non-scholarly citations, Earned media mentions, Social media mentions, and Usage. Row 1: Research productivity and scope, Definition, Data source. Row 2: Publications by year, The cumulative number of publications produced by year, with year defined as the calendar year when the manuscript was first made publicly available, electronically or in print, Scopus, PubMed. Row 3: Publications by study setting, The cumulative number of publications by study setting, with setting categories constructed according to RADx-UP internal operational definitions. Not all publications had a designated setting. For instance, systematic reviews and letters to the editor were not assigned a setting., Structured coding. Row 4: Publications by focal population, The cumulative number of publications reporting results of studies focused on that population, categorized based on NIH definitions of underserved and vulnerable populations[27]. Publications could have more than one focal population or no focal population., Structured coding. Row 5: Count of publications by study type, The cumulative number of publications categorized by study type, with definitions of study types developed through reference to established definitions for qualitative and quantitative research methods[28-30]., Structured coding. Row 6: Co-author affiliations, The listed affiliation for publication co-authors, categorized according to an internally developed taxonomy that was vetted with the RADx-UP program team. Categories were: Community: Organization with direct relationships with the target population, serves target population at the community level; School: Formal school settings for children from pre-primary to secondary school, includes daycare, pre-school, and K-12 school; Government: Government organization at the local, state or federal level, including health department or other governmental organization; Professional: Professional groups which include professional associations, consulting groups, contract research organizations, for-profit businesses, insurance companies; Healthcare: Organization with a primary scope of work that includes providing health services to individuals, includes non-research hospital units and clinics; Academic: Conducts research within a university or hospital. When needed, a hierarchy was applied, with the highest-ranking category being selected when more than one affiliation was listed: 1) community, 2) school, 3) government, 4) professional, 5) healthcare, 6) academic., Structured coding. Row 7: Initial translational impact,, . Row 8: Scholarly citations, The number of times a publication has been cited in other scholarly publications indexed in the Scopus database., Scopus. Row 9: RCR, A publication’s average number of citations per year is divided by the citation rate of NIH-funded papers in the same field and year. The co-citation network, which includes all the publications cited alongside the scholarly publications, defines the field for each scholarly publication. A paper with an RCR of 1.0 has received the same number of citations per year as the typical (median) NIH-funded paper in its field. A paper must be published for at least a year or have accrued at least five citations to calculate an RCR[26]., iCite. Row 10: Captures, Count of saves, archives, or other actions allowing someone to come back to a publication later. Examples include bookmarks, code forks, favorites, followers, readers, watchers, exports/saves and subscribers. Captures can be a leading indicator of future citations[31]., PlumX. Row 11: Non-scholarly citations, The number of times a publication has been cited in non-scholarly documents such as patent applications, clinical practice guidelines, or legislative filings or position papers. Complementing the number of scholarly citations alone, this metric gives a sense for societal impact[31]., PlumX. Row 12: Earned media mentions, Number of times a publication is cited in news or non-scholarly communication channels. Examples include blog mentions, news media mentions, reviews, and Wikipedia references. Mentions are an indicator that people are engaging with the research[31]., PlumX. Row 13: Social media mentions, The number of times a publication is referenced on Facebook. Examples include comments, shares, likes, ratings, and recommendations that reference the research. This metric is an indicator of grass-roots attention research is receiving[31]., PlumX. Row 14: Usage, The number of interactions with a publication indicating real-time use, including abstract views, clicks, downloads, full text views, library holdings, and video plays[31]., PlumX.

Navigate back to Table 1..

## Table 2. Long description

A table with four rows and three columns describing social network measures, their definitions, and levels. The columns are labeled Measure, Definition, and Level. Row 1: Node degree, The number of direct connections a node (i.e., member) has. In this context it is the number of co-authors an author has across their publications., Individual. Row 2: Components, A cluster of nodes with connections to each other and no connections to nodes outside their cluster., Network. Row 3: Community representation, Percent of components contain co-authors who either represent community organizations or have strong affiliations with one., Network. Row 4: Power centrality, A common measure of influence in social network analysis where the centrality of individuals changes based on their connection to other nodes and their respective influence in the network. Positive values of power centrality imply that members of the network are connected to relatively central members occupying a position important to the flow of information. Conversely, negative values reflect connection to members who are relatively less central., Individual.

Navigate back to Table 2..

## Table 3. Long description

A table summarizing the characteristics of publications produced by the RADx-UP initiative through June 2024. The table has 13 rows and 3 columns. The columns are labeled ʼPublication characteristicsʼ, ʼCountʼ, and ʼ(% of publications)ʼ. The rows are grouped into four categories: Publication year, Setting, Focal population, and Study type. Each row provides specific details under these categories. For Publication year, the data spans from 2020 to 2024, with counts and percentages of publications for each year. For Setting, the data includes various locations such as School, Residential care facility, Community health center, In-home, Hospital, and Prison or jail, with corresponding counts and percentages. For Focal population, the data includes groups like Hispanic or Latino, Children or adolescents, Black or African American, Low socioeconomic status, Rural, People who inject drugs, American Indian, Older adults, Asian Americans, Pacific Islanders, People who are incarcerated, Immigrants or refugees, Alaska Natives, People experiencing homelessness, Native Hawaiian, Pregnant people, and Sexual and gender minorities, each with their respective counts and percentages. For Study type, the data includes Original research, Quantitative, Qualitative, Mixed methods, Editorial, Literature review, and Clinical case study, with counts and percentages for each type. The table provides a detailed breakdown of the scope and focus of the publications produced by the RADx-UP initiative.

Navigate back to Table 3..

## Table 4. Long description

A table summarizing translational impact metrics for publications by the RADx-UP initiative. The table has two columns: Metric and Mean (range). The Metric column lists various bibliometrics and altmetrics, while the Mean (range) column provides corresponding values. The table includes 11 rows of data. Row 1: Total citation count (n = 328), 2,641 (0-91). Row 2: Relative citation ratio (n = 300), 2.07 (0.00-12.94). Row 3: Captures, 8,208. Row 4: Downloads, 122. Row 5: Abstract views, 115. Row 6: Policy citations, 90. Row 7: News, 736. Row 8: Blogs, 25. Row 9: Facebook, 1,971.

Navigate back to Table 4..

## Table 5. Long description

The table presents network analysis metrics for the RADx-UP publications co-author network, overall and for community partners, across the years 2022, 2023, and 2024. It consists of 6 rows and 4 columns, including the headers. The columns are labeled Metric, 2022, 2023, and 2024. The rows are labeled Network overall, Mean node degree, # of components, Community partners, Representation in components, Mean node degree, and Power centrality. Row 1: Metric, 2022, 2023, 2024. Row 2: Network overall,, , . Row 3: Mean node degree, 14.37, 18.04, 26.02. Row 4: # of components, 28, 34, 29. Row 5: Community partners. Row 6: Representation in components, .18, .37, .41. Row 7: Mean node degree, 13.83, 25.17, 19.69. Row 8: Power centrality, -0.96, -0.75, -0.37. The table shows an increase in the number of components over the years. Mean node degree initially increased but then decreased. The representation in components for community partners also show an increasing trend. The power centrality values become less negative over the years.

Navigate back to Table 5..
